# Psychological intervention on COVID-19: A protocol for systematic review and meta-analysis

**DOI:** 10.1097/MD.0000000000020335

**Published:** 2020-05-22

**Authors:** Gu Renjun, Li Ziyun, Yan Xiwu, Wei Wei, Gu Yihuang, Zhang Chunbing, Sun Zhiguang

**Affiliations:** a The First School of Clinical Medicine; b The Third School of Clinical Medicine; c Acupuncture and Tuina School, Nanjing University of Chinese Medicine; d Jiangsu Province Hospital of Chinese Medicine; e The Second School of Clinical Medicine, Nanjing University of Chinese Medicine, Nanjing, Jiangsu Province, China.

**Keywords:** COVID-19, meta-analysis, psychological intervention, systematic review

## Abstract

**Introduction:**

COVID-19 is novel coronavirus infection in 2019. Many reports suggested that psychological intervention is playing a positive role in COVID-19 treatment, but there is no high-quality evidence to prove its effects. This paper reports the protocol of a systematic review and meta-analysis to clarify effectiveness of psychological intervention during the treatment of COVID-19.

**Methods and analysis:**

The following electronic databases will be used by 2 independent reviewers: Web of Science, Embase, Cochrane Library, PubMed, Chinese Biomedical Literature Database, Chinese National Knowledge Infrastructure, Chinese Scientific Journal Database, Wan fang Database, ClinicalTrials, WHO Trials, and Chinese Clinical Trial Registry. The randomised controlled trials of psychological intervention on COVID-19 will be searched in the databases by 2 researchers independently. Clinical recovery time and effective rate will be assessed as the primary outcomes. Changes of patients physical condition (1. Time until COVID-19 RT-PCR negative in upper respiratory tract specimen; 2. Time until cough reported as mild or absent; 3. Time until dyspnea reported as mild or absent; 4. Frequency of requiring supplemental oxygen or non-invasive ventilation; 5. Frequency of requiring respiratory; 6. Incidence of severe cases; 7. Proportion of re-hospitalization or admission to ICU; 8. All-cause mortality; 9. Frequency of seriously adverse events) and changes of psychological condition (such as: SRQ-20, PHQ-9, GAD-7, Hamilton Depression Scale, Hamilton Anxiety Scale) will be assessed as the secondary outcomes. For dichotomous outcomes, such as effective rate, data will be expressed as risk ratio (RR) with 95% confidence intervals (CIs). For continuous outcomes, weighted mean differences (WMD) or standardized mean differences (SMD) will be calculated. Fixed effect model will be used for evaluating efficiency. Considering clinical heterogeneity, random effect model will be used for continuous outcomes.

**Results:**

Relevant studies will be used to evaluate whether psychological intervention is effective for COVID-19.

**Conclusion:**

This study will provide reliable evidence for psychological intervention on COVID-19.

**PROSPERO registration number:**

CRD42020178699

## 1 Introduction

COVID-19 is a new acute infectious disease caused by Corona Virus of severe acute respiratory syndrome coronavirus 2.^[1]^ World Health Organization has listed it as a Public Health Emergency of International Concern. People of all ages are vulnerable to infections and it will have a negative impact on psychological health.^[2]^ The symptoms of COVID-19 patients mainly include fever, fatigue, cough, shortness of breath/respiratory distress, etc.,^[3]^ Chest CT with pneumonia.^[4]^ COVID-19 is now spreading rapidly and have negative impact on mental health.^[5]^ However, there is no effective treatment for this disease currently. Symptomatic treatment and supportive care are considered as the major treatment methods.^[6]^ Therefore, psychological intervention is being considered as adjuvant therapy to provide more help for COVID-19 patients.

Psychotherapy uses psychological methods to educate and treat patients. It can eliminate physical symptoms and improve mental health.^[7,8]^ COVID-19 may cause public panic and mental stress.^[9]^ Some COVID-19 patients feel anxious and difficult to reintegrate into society. In addition, quarantine has been used in COVID-19 outbreak.^[10]^ It will have negative emotions such as fear, depression, boredom, etc.^[11]^ Using psychological intervention will reduce psychological stress and help to integrate COVID-19 patients into society. What's more, it will relieve the patients anxiety and prevent immunity decline.^[12,13]^ However, most of the clinical trials provided insufficient evidence due to the small sample sizes. It lacks sufficient evidence to prove the effectiveness of psychological intervention on COVID-19 patients. Therefore, we will conduct a systematic review and meta-analysis to provide reliable evidence for psychological intervention on COVID-19.

## 2 Methods

### 2.1 Study registration

This protocol refers to the guide book of Preferred Reporting Items for Systematic Reviews and Meta-Analyses Protocols (PRISMA-P)^[14]^ and it was registered in PROSPERO (CRD42020178699).

### 2.2 Search strategy

The following electronic databases will be used by 2 independent reviewers: Web of Science, Embase, Cochrane Library, PubMed, Chinese Biomedical Literature Database, Chinese National Knowledge Infrastructure, Chinese Scientific Journal Database, Wan fang Database, ClinicalTrials, WHO Trials and Chinese Clinical Trial Registry. Reference lists of articles, grey literature, and conference proceedings will also be searched. Languages of the publications will be limited to English and Chinese.

PubMed literature search as following:

#1. Search “COVID-19” [Mesh]

#2. Search (((((((((((((((2019 novel coronavirus infection) OR COVID19) OR coronavirus disease 2019) OR coronavirus disease-19) OR 2019-nCoV disease) OR 2019 novel coronavirus disease) OR 2019-nCoV infection) OR Wuhan coronavirus) OR Wuhan seafood market pneumonia virus) OR COVID19 virus) OR COVID-19 virus) OR coronavirus disease 2019 virus) OR SARS-CoV-2) OR SARS2) OR 2019-nCoV) OR 2019 novel coronavirus

#3. Search #1 OR #2

#4. Search “Psychotherapy”[Mesh]

#5. Search ((((((((((((Psychotherapies) OR Psychotherapists) OR Psychotherapist) OR Clinical Psychotherapists) OR Clinical Psychotherapist) OR Psychotherapist, Clinical) OR Psychotherapists, Clinical) OR Schema Therapy) OR Schema Therapies) OR Therapies, Schema) OR Therapy, Schema) OR Logotherapy) OR Logotherapies

#6. Search #4 OR #5

#7. Search # 3 AND #6

### 2.3 Study selection

#### 2.3.1 Type of study

Randomized Controlled Trials (RCTs) will be adopted. If some experiments do not explain randomization, the literature will be considered as high risk in random sequence generation.

#### 2.3.2 Inclusion criteria

1. Participants could be of any age, sex or ethnic origin, and the patient has to be diagnosed with COVID-19.
2. Published literature.
3. Intervention measures: Interventions using psychological intervention as a main variable. Any comparisons between a combined therapy of psychological intervention and other interventions and a therapy of solely using other interventions are also included.
4. The control group will be no-treatment, regular treatment or nursing.

#### 2.3.3 Exclusion criteria

1. Literatures published repeatedly by the same author or with duplicate data;
2. Literatures with less than 10 samples in experimental group or control group.

#### 2.3.4 Outcomes and prioritization

The primary outcomes will include mean clinical recovery time (hours) and Effective rate. The clinical recovery time is defined as the time from initiation of psychological intervention (experimental or control group) on normalization of fever, respiratory rate, and oxygen saturation, and alleviation of cough, sustained for at least 72 hours. Effective rate is based on whether psychological intervention can improve patients condition such as fever, respiratory rate, and oxygen saturation, and cough. Additional outcomes of patients condition are as follows: Changes of patient's physical condition (1. Time until COVID-19 RT-PCR negative in upper respiratory tract specimen; 2. Time until cough reported as mild or absent; 3. Time until dyspnea reported as mild or absent; 4. Frequency of requiring supplemental oxygen or non-invasive ventilation; 5. Frequency of requiring respiratory; 6. Incidence of severe cases; 7. Proportion of re-hospitalization or admission to ICU; 8.All-cause mortality; 9.Frequency of seriously adverse events) and changes of psychological condition (such as: SRQ-20, PHQ-9, GAD-7, Hamilton Depression Scale, Hamilton Anxiety Scale). If a new suitable form is found in the literature search, it will be taken into consideration.

### 2.4 Data collection

#### 2.4.1 Data management

Endnote X9.3 will be used to manage the search results and perform screening. The statistical calculation process will be completed by RevMan5.2 software, and the sensitivity analysis will be completed by Stata/SE 15.1 software.

#### 2.4.2 Data extraction

**Figure 1:**
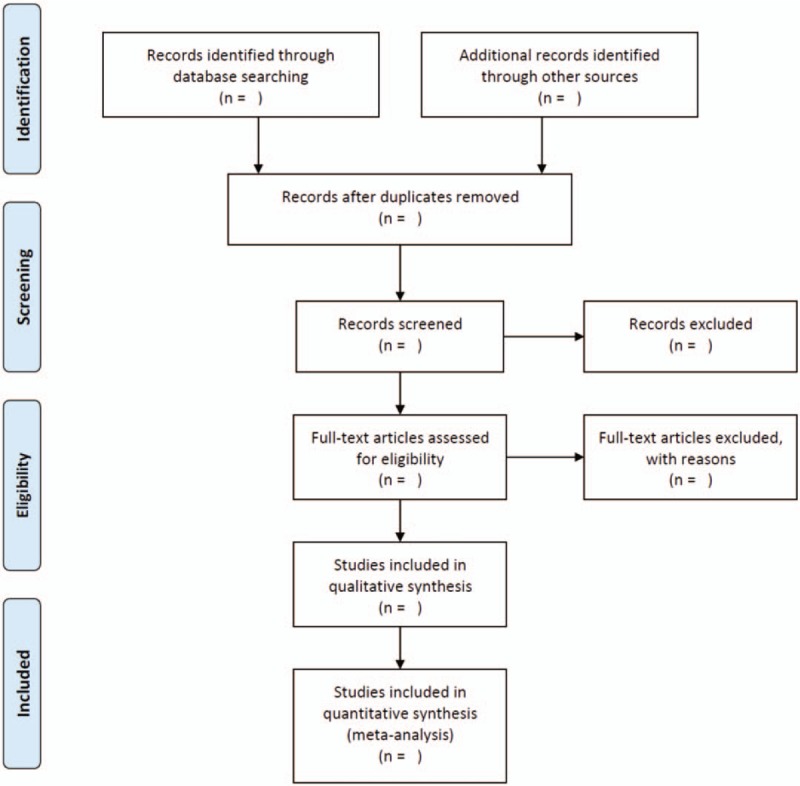
PRISMA flow diagram of the study process. PRISMA, Preferred Reporting Items for Systematic review and Meta-Analysis.

According to the inclusion and exclusion criteria, 2 review authors will independently scan the articles and investigate the potentially eligible articles as full text. If disagreement exists between the authors, a third expert or the whole group members will join the discussion. Two main authors independently collected data on study characteristics (including the first author, year, patients condition, observation group, control group, the main points, course of psychological intervention, and the main outcomes) using a standardized data extraction form for eligible trials. The PRISMA flow chart shows the process of study selection (Fig. 1).

#### 2.4.3 Risk of bias assessment

According to the risk of bias risk assessment tool of Cochrane,^[15]^ 2 authors independently assessed the bias risk of the included literature, and the opinions will be discussed when they are different. Bias risk will be assessed from 7 items: Random sequence generation, Assignment concealment, Blinding of participants and personnel, Blinding of outcome assessment, Incomplete outcome data, Selective reporting, and other bias. RevMan software is an evaluation tool provided by Cochrane, which is used to evaluate the risk bias of literature quality. It analyzes the quality of literatures visually, using green, yellow and red colors and “+”, “-”, “?” “ The symbols indicate ”low risk bias“, ”high risk bias“ and ”unclear“ to evaluate literatures 1 at a time.

#### 2.4.4 Dealing with missing data

We will contact authors with missing or incomplete data in the included articles by email. However, if the missing data cannot be obtained, then the study will be excluded from the analysis.

### 2.5 Statistical analysis

#### 2.5.1 Data synthesis

The statistical package (RevMan) will be used for data analysis. *P* value and *I*^2^ statistic will be used to test heterogeneity between trial results. Heterogeneity will be considered when more than 2 articles are included. If the *I*^2^ > 50%, the random effect model will be applied according to the Clinical heterogeneity.

For dichotomous outcomes, such as effective rate, data will be expressed as risk ratio (RR) with 95% confidence intervals (CIs), and differences between the intervention and control groups will be assessed. Continuous outcomes, such as mean clinical recovery time (hours), weighted mean differences (WMD) or standardized mean differences (SMD) will be calculated. In addition, the fixed effect model will be used for efficiency. The random effect model will be used for continuous outcomes in light of clinical heterogeneity. Forest plots will be used for data presentation.

#### 2.5.2 Subgroup analysis

If there is significant heterogeneity in the included trials, subgroup analysis will be carried out. According to subject characteristics (e.g., severity of COVID-19, age, gender, and so on), subgroup analysis will be carried out according to the data retrieved.

#### 2.5.3 Sensitivity analysis

If there is still significant heterogeneity in the included trials after subgroup analysis, Sensitivity analysis will be performed to assist exploring the source of heterogeneity. It will be carried out by deleting each study at a time, and other studies will be analyzed to estimate whether a single study would have a significant impact on the results.

### 2.6 Ethics and dissemination

Ethical approval will not be needed because no primary data will be used in this protocol. The results of the systematic review focus on exploring the effectiveness of psychological intervention on COVID-19.

## 3 Discussion

In the prevalence of COVID-19, there is no effective medication. Many reports suggested that more attention should be paid to psychological intervention on COVID-19.^[2,16]^ If psychological intervention as complementary treatment can improve the symptoms of COVID-19, it will bring benefits for COVID-19 patients. This analysis aims at deeply understanding the involvement of psychological intervention in COVID-19 adjuvant therapy as well as looking forward to providing reference for clinical treatment.

Strengths and limitations will be highlighted during identifying evidence. The data extraction and risk of bias assessment will be completed by 2 researchers independently, which will provide accurate evidence for psychological intervention. In addition, this analysis will solve the hot research topic of COVID-19 and provide reference for clinical guideline. Limitations will mainly originate from different clinical situation and different basic treatment on COVID-19 patients. It may lead to high heterogeneity and lower the quality of the evidence. However, subgroup analysis and sensitivity analysis will be used to overcome these heterogeneities in the meta-analysis. The results of this meta-analysis may help to establish a better approach to treating COVID-19 and to provide reliable evidence for application of psychological intervention.

## Author contributions

**Conceptualization:** Renjun Gu, Chunbing Zhang, Zhiguang Sun.

**Investigation:** Renjun Gu, Ziyun Li, Xiwu Yan, Wei Wei.

**Methodology:** Renjun Gu, Ziyun Li, Yihuang Gu, Zhiguang Sun.

**Project administration:** Renjun Gu, Yihuang Gu, Chunbing Zhang, Zhiguang Sun.

**Writing – original draft:** Renjun Gu, Ziyun Li, Xiwu Yan, Wei Wei.

**Writing – review & editing:** Renjun Gu, Yihuang Gu, Chunbing Zhang, Zhiguang Sun.
